# Impact of Affordable Care Act provisions on the racial makeup of patients enrolled at a Deep South, high-risk breast cancer clinic

**DOI:** 10.21203/rs.3.rs-3359906/v1

**Published:** 2023-10-24

**Authors:** Jillian Tinglin, M. Chandler McLeod, Courtney P. Williams, Meghan Tipre, Gabrielle Rocque, Andrew B. Crouse, Helen Krontiras, Lily Gutnik

**Affiliations:** The University of Alabama at Birmingham Heersink School of Medicine; The University of Alabama at Birmingham Department of Surgery; UAB DOPM: The University of Alabama at Birmingham Division of Preventive Medicine; University of Pittsburgh Department of Medicine; UAB DOM: The University of Alabama at Birmingham Department of Medicine; The University of Alabama at Birmingham Hugh Kaul Precision Medicine Institute; UAB Surgery: The University of Alabama at Birmingham Department of Surgery; UAB Surgery: The University of Alabama at Birmingham Department of Surgery

**Keywords:** Breast cancer, high-risk, preventive care, Affordable Care Act, disparities, African American, race

## Abstract

**Purpose:**

Black women are less likely to receive screening mammograms and are at a higher lifetime risk for developing breast cancer compared to their White counterparts. Affordable Care Act (ACA) provisions decreased cost sharing for women’s preventive screening, potentially mitigating screening disparities. We examined enrollment of a high-risk screening program before and after ACA implementation stratified by race.

**Methods:**

This retrospective, quasi-experimental study examined the ACA’s impact on patient demographics at a high-risk breast cancer screening clinic from 02/28/2003–02/28/2019. Patient demographic data were abstracted from electronic medical records and descriptively compared in the pre- and post-ACA time periods. Interrupted time series (ITS) analysis using Poisson regression assessed yearly clinic enrollment rates by race using incidence rate ratios (IRR) and 95% confidence intervals (CI).

**Results:**

2,767 patients enrolled in the clinic. On average, patients were 46 years old (SD, ± 12), 82% were commercially insured, and 8% lived in a highly disadvantaged neighborhood. In ITS models accounting for trends over time, Prior to ACA implementation, White patient enrollment was stable (IRR 1.01, 95% CI 1.00–1.02) while Black patient enrollment increased at 13% per year (IRR 1.13, 95% CI 1.05–1.22). Compared to the pre-ACA enrollment period, the post-ACA enrollment rate remained unchanged for White patients (IRR 0.99, 95% CI 0.97–1.01) but decreased by 17% for Black patients (IRR 0.83, 95% CI 0.74–0.92).

**Conclusion:**

Black patient enrollment decreased at a high-risk breast cancer screening clinic post-ACA compared to the pre-ACA period, indicating a need to identify factors contributing to racial disparities in clinic enrollment.

## Introduction

Black women are less likely to undergo breast cancer screening, and more likely to present with more advanced breast cancers at initial mammographic screening when compared to other racial groups [1]. Differences in access to screening services and persistent barriers to the cost of screening including insurance status and socioeconomic status (SES) have been explored as agents contributing to these discrepancies [2]. Average risk women have an approximate 13% lifetime risk for developing breast cancer, whereas women considered at high-risk have a 20% or greater lifetime risk. Black women have a higher lifetime risk for developing breast cancer compared to White women [2]. Uptake of certain clinical preventive services has been historically low among high-risk women, and further aggravated by racial disparities [3, 4].

Defined by the five basic tenets of availability, accessibility, accommodations, affordability, and acceptability, access is one common barrier for health disparities existing at the state and national level [5]. To improve access to health care, the Affordable Care Act (ACA) was a landmark bill that increased the overall percentage of insured individuals in the U.S. It also aimed to improve access to preventive services by decreasing cost sharing insured women, including decreased cost for screening mammograms every 2 years for women over the age of 40 [6]. Studies have shown increased rates in the uptake of breast cancer screening services since the passage of the ACA as facilitated by increased access to insurance coverage via Medicaid expansion for lower income individuals [7]. Although many studies show that overall breast cancer screening rates increased in Medicaid expansion states, non-expansion states also experienced increased uptake of breast cancer screening services [8, 9]. In the context of non-expansions states like Alabama where higher levels of poverty exist amongst its Black residents, the ACA may have influenced an increased uptake of women’s preventive services in the post-expansion period [10].

Considering the potential impact of the ACA on increasing affordability and therefore accessibility to women’s preventive screening, especially in states where higher proportions of Black residents live in poverty, we evaluated the change in clinic enrollment and racial make-up at the University of Alabama at Birmingham’s Preventive Care Program for Women’s Cancer (UABPC) before and after ACA implementation. The UABPC is a high-risk women’s cancer prevention clinic founded in 2001, housed in a tertiary academic medical center. The UABPC offers annual high-risk breast cancer screening including MRI, mammogram, clinical breast exam, access to screening and prevention research trials, comprehensive breast cancer risk assessment, and genetic counseling and testing. We hypothesized that there would be an increase in the proportion of Black patient enrollment within the UABPC post-ACA when compared to pre-ACA levels.

## Methods

### Study design and patient population:

This retrospective, quasi-experimental study used an interrupted time series approach to examine the impact of ACA provisions upon the racial makeup of the UABPC. Women aged 20–82 years seen for their initial UABPC appointments between February 2003 and February 2019 who consented to be in the UABPC database were included in this study. Exclusion criteria included primary residence outside of Alabama. IRB approval was subsequently obtained (IRB-030409006).

### Outcomes: Racial composition of patients seen at the UABPC

The primary outcome for this study was change in the racial composition of patients seen at the UABPC clinic pre-versus post-implementation of ACA provisions. Racial composition of women seen at the UABPC was calculated using the proportional count of (1) White, or (2) black or African American patients seen in comparison relation to all patients seen at the UABPC by month. Proportions of Asian, Hispanic, Other, and Unknown categories were not evaluated in the ITS analysis estimates due to small sample sizes. However, their enrollment was still account for when calculating proportions of White and Black patients.

### Exposure: Implementation of ACA provisions

The exposure of interest was implementation of ACA provisions, effective January 1, 2014. We examined monthly pre-intervention time periods prior to ACA implementation (February 2003-December 2013) monthly post-intervention time periods (January 2014-February 2019).

### Covariables:

Patient sociodemographics abstracted from the UABPC database and electronic health record included age at enrollment, race (White, Black, or African American, other [Hispanic, Native American, unknown]), insurance status (private/commercial, Medicaid, Medicare, other [self-pay, Tricare, charity care, other]), and home address. Patient addresses were used to identify neighborhood disadvantage on the county level, as measured by the Area Deprivation Index (ADI). ADI was scored from 1–100%, with higher percentages representing higher neighborhood disadvantage. For this study, Alabama census tract ADI values for 2015 and 2020 were averaged by county and then linked to patients’ records using patient county and enrollment date. Patients who enrolled before 2017 were assigned their county’s mean 2015 ADI and patients who enrolled in 2017 or later were linked to their county mean 2020 ADI. ADI was dichotomized into high (> 85%) and low (≤ 85%) neighborhood disadvantage [11].

### Analysis:

Descriptive statistics, including means and standard deviations for continuous variables and frequencies for categorical variables, were calculated for patient sociodemographic data and compared pre- and post-ACA implementation. An interrupted time series analysis using a quasi-Poisson model with log link and an offset for the log of the total number of enrollments was used to assess our primary outcome using the following level-change regression model [12]:

$$log\left(Y_{t}\right)==log\left(n\;total\;\left.I_{t}\right)+\beta_{0}+\beta_{1}{\;}^{*}Time\;+\beta_{2}{\;}^{*}\left(ACA\;Implementation\;\right)+\beta_{3}{\;}^{*}(Time\;*\;ACA\;Implementation)\right.$$

In the described basic model, the coefficient $\beta_{0}$ estimates the expected log number of patients in Q1 2003 among all patients, and $\beta_{1}$ estimates the trend of log number of patients seen per year prior to ACA implementation. The coefficient $\beta_{2}$ is the immediate effect of ACA provisions on the log number of patients enrolled after the intervention, and $\beta_{3}$ is the change in the trend of the log number of patients seen per year post-ACA implementation relative to the pre-ACA period. The offset consists of the natural log of the total number of enrollments for the time period. While enrollments were aggregated on a quarterly basis, coefficients were evaluated for 1-year change. Exponentiation of coefficients provides for the corresponding estimated incident rate ratios. Separate models were constructed to assess enrollment rates for White and Black or African American patients. To account for potential confounding by socioeconomic status, sensitivity analyses were performed for each model adjusting for neighborhood disadvantage as measured by ADI. ADI was incorporated into models by calculating the proportion of patients who had ADI values greeter than 85% in each quarter. Analysis was performed in R (version 4.2.1, 2022) and significance was assessed at p-value < 0.05.

## Results

### Study sample

Of the 2,767 women seen at the UABPC, mean age was 46 years (SD 13), 13% were Black or African American, 82% were commercially insured, and 8% lived in a highly disadvantaged neighborhood (Table 1). Compared to the time period before ACA, patients seen after implementation of ACA provisions were younger (mean age 45 vs. 48) .

### Racial trends for patients seen at the UABPC

ITS analysis estimates indicated rates of White patient enrollment at the UABPC did not increase or decrease during the pre-(IRR 1.01 95% CI 1.00–1.02) or post-ACA implementation periods (IRR 1.00 95% CI 0.98–1.02) (Table 2). Conversely, rates of Black patient enrollment increased by 13% per year pre-ACA implementation (IRR 1.13, 95% CI 1.05–1.22). Immediately post-ACA implementation, the rate of White and Black patient enrollment did not change significantly (IRR 1.34, 95% CI 0.93–1.94 and IRR 0.96, 95% CI 0.89–1.03). However, post-ACA implementation, rate of Black patient enrollment decreased by 17% per year (IRR 0.83, 95% CI 0.74–0.92) compared to the pre-ACA rate of Black patient enrollment. In sensitivity analyses adjusting for neighborhood disadvantage in the ITS analysis, similar trends were observed (Supplemental Table 1).

## Discussion

Our results demonstrate that, in the five-year period following ACA implementation, Medicaid expansion and implementation of cost-sharing preventive services for average risk women in the U.S., yearly rates of Black and White patient enrollment remained stable. However, in the context of the effect of ACA implementation on the rate of patient enrollment relative to the pre-ACA period, our results showed that rates of White patient enrollment remained consistent throughout pre and post-ACA implementation periods. Rates of Black patient enrollment, however, decreased in the post-ACA time period relative to the pre-ACA time period.

Further research must be done to elucidate the additional factors associated with the significant decrease in Black patient enrollment following implementation of expanded women’s preventive services by the ACA compared to the pre-ACA implementation period. For example, publicization of the UABPC’s existence and its referral process may be optimized to improve access. Knowledge of the UABPC is largely spread through internal providers within the UAB healthcare system by word of mouth rather than a standardized referral process. Providers external to the UAB system are less likely to be aware of the clinic thus less likely to refer patients. Additionally, most patients are self-referrals and are aware of the clinic via spoken communication. This is another opportunity to examine racial disparities.

ACA implementation and Medicaid expansion was enacted as a path to increase the percentage of the insured in the U.S., especially those in low-income communities. Historically, more African Americans live in poverty or at a lower socioeconomic status when compared to their White counterparts [13]. Although the ACA was targeted at this community and other communities at lower socioeconomic status, we noted a decrease in the enrollment of Black patients during the post-expansion period. In exploring the possible reasons for this downward trend in enrollment, it is possible that the historical distrust of the healthcare system within the Black community could have adversely influenced Black patient enrollment in the UABPC. This pervasive and warranted distrust is vested in the historical mistreatment and abuse of African Americans by the U.S. healthcare system, with levels of mistrust higher amongst those African Americans that are known descendants of the enslaved [14]. In a community that has already been marginalized by the U.S. healthcare system, it is possible that patients of color who had a dissatisfying experience within the UABPC could have discouraged their community members from seeking further care through the clinic. Furthermore, in certain minority communities, health concerns and hardships are sometimes considered culturally taboo, and therefore it can be difficult to establish a thorough history and encourage self-advocacy in seeking preventive care through the UABPC [15]. Alternatively, it is possible that during the post-expansion period, there was a decrease in the number of providers within and outside the UAB system that served a greater number of African American patients. It can be equally challenging for a patient who is already distrustful of the healthcare system to reestablish care with a provider with whom they do not have an established history. Because of these potential environmental shifts that can contribute to patient care and could cause a downtrend in UABPC patient enrollment, further investigation is warranted to understand the referral process to the UABPC and communities it predominantly serves.

Within the community, primary care providers such as family medicine physicians and Obstetrician Gynecologists primarily conduct breast cancer risk assessments to estimate women’s lifetime risk for breast cancer based on a variety of modifiable and non-modifiable risk factors [16]. The variety of available risk assessment tools can also lead to their underutilization, resulting in patients’ who are unsure of their own risk status for breast cancer [17]. Community physicians may opt to manage these patients themselves rather than referring to a breast cancer specialist in the absence of palpable mass or breast cancer diagnosis. Additionally, statewide, physicians in the community may not be aware of the existence of the UABPC, and therefore not refer their patients.

Uninsurance or underinsurance is associated with other social determinants of health such as belonging to a minority racial group, having a lower educational status, or earning a lower wage [18, 19]. These characteristics predispose patients to a decreased potential of upward social mobility, influencing their overall socioeconomic status and directly obstructing access to preventive care or appropriate screening for their high risk status. Although non-Medicaid expansion states like Alabama did not receive additional funding to expand women’s preventive services, many non-Medicaid expansion states still experienced an increase in insurance utilization following Medicaid-expansion, likely due to the increased awareness of insurance accessibility and importance of preventive healthcare initiatives popularized by the ACA [18]. In light of the central drive for Medicaid-expansion, if Alabama were to expand Medicaid, its citizens could experience increased insurance access and access to breast cancer screening modalities, incur lower incidences of late-stage breast cancer diagnoses, and become a more health-informed population [20].

A notable limitation in this study was that the initial provisions within the ACA decreased cost sharing for women’s preventive services for women at average risk for developing breast cancer, rather than high-risk. Despite this, we felt trends reflected in the average risk community, such as increased mammography screening, would be mirrored in the high-risk community. In addition to this limitation, we acknowledge that further investigation into the clinic referral patterns of the UABPC may yield a better understanding of the decreased enrollment of Black patients in the UABPC during the post-ACA period. Additionally, our study reflects the trends of enrollment for those patients who were able to access the UABPC either through self or physician referral. Therefore, our trends may not be incorporating those patients that are the most under-represented which would suggest a more notable inequity in access to high-risk preventive services in the post-expansion period.

## Conclusions

Our data we demonstrated there was a statistically significant decrease in Black patient enrollment rate in the post-ACA time period relative to the pre-ACA time period. Racial disparities persist despite nationwide efforts to improve access to preventive healthcare, as is demonstrated in our study within a unique clinic.

Multi-level interventions addressing racial disparities in the high-risk breast cancer community are needed to increase equity for access of preventive services. Through the increased publicization of preventive care clinics such as the UABPC as well as a greater understanding of the referral process of the UABPC, the cost-related barriers to accessing these services can be lowered and the gaps in healthcare accessibility further narrowed between White and Black patients.

## Figures and Tables

**Figure 1:**
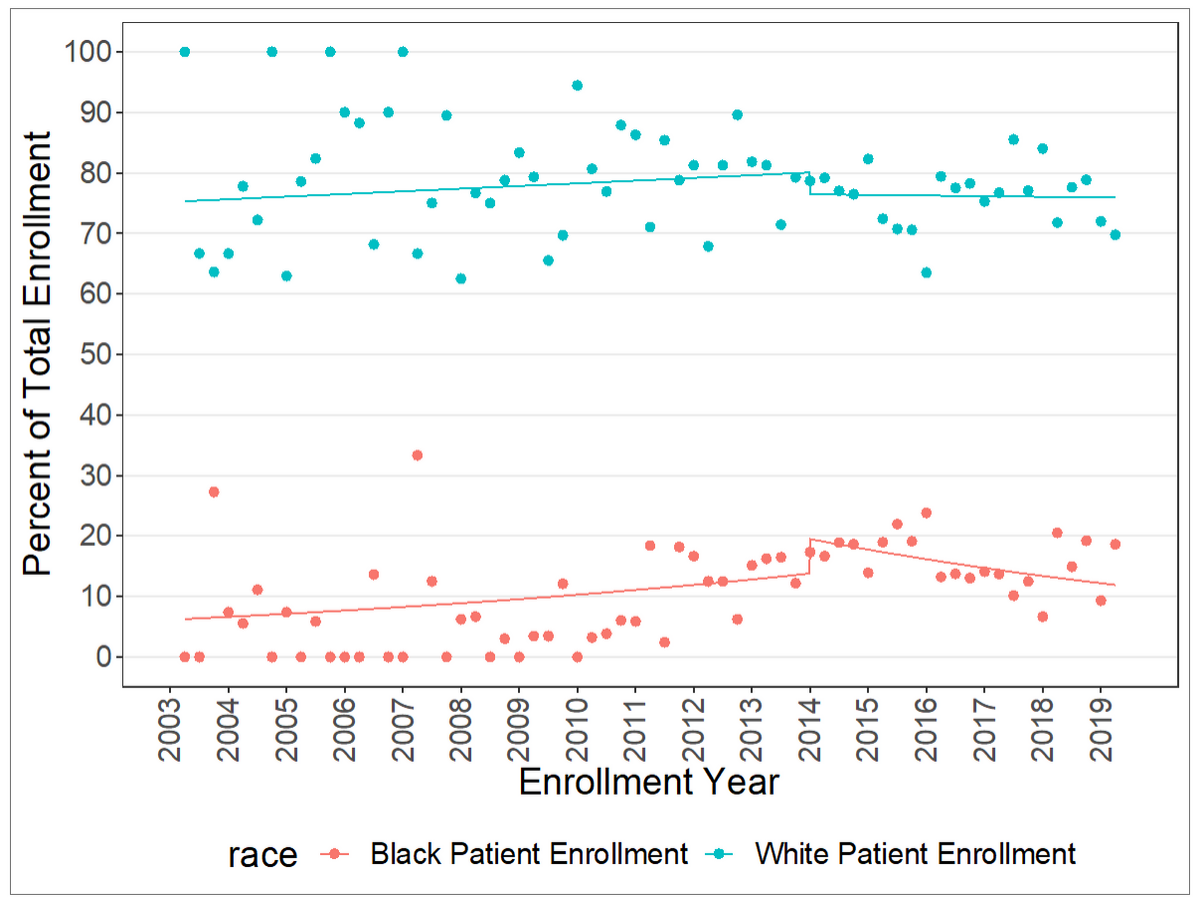
Rates of Enrollment to UAB Preventive Care Program for Women’s Cancer by race from, Q1 2003 to Q1 2019.

**Table 1.** Patient sociodemographic characteristics by time seen at the UAB Preventive Care Program for Women's Cancer (N = 2737).

	Total N (%) (N = 2767)	Pre-Affordable Care Act Provisions N (%) (n = 1285)	Post-Affordable Care Act Provisions N (%) (n = 1482)	P-value
Age (mean, SD)	46 (13)	48 (12)	45 (13)	.001
Race				<.001
Black/African American	362 (13)	129 (10)	233 (16)	
White	2134 (78)	1006 (79)	1128 (77)	
Other*	248 (9)	147 (12)	101 (7)	
Insurance status				.16
Commercial	2172 (82.1)	944 (80.6)	1228 (83.4)	
Medicare	312 (11.8)	146 (12.5)	166 (11.8)	
Medicaid	56 (2.1)	26 (2.2)	30 (2.0)	
Other**	104 (3.7)	55 (4.3)	49 (3.3)	
Missing***	123	114	9	
Neighborhood disadvantage status				.28
High disadvantage (ADI >85)	132 (8.3)	64 (7.7)	68 (9.0)	
Low disadvantage (ADI < 85)	1452 (91.7)	763 (92.3)	689 (91.0)	
Missing	1183	458	725	

SD = standard deviation

* Asian, Native American, Hispanic, Other, Unknown

** Self Pay, Tricare, Charity, Agency

*** Not included in p-value calculation

**Table 2.** Interrupted time series quasi-Poisson model-estimated rates of UAB Preventive Care Program for Women's Cancer enrollment by White and Black race (n = 65 quarters).

	Rate of White enrollment (comparing change in rate from pre-ADA to post-ADA)			Rate of White patient enrollment (evaluating change in rate in pre and post ADA periods)			Rate of Black/African American enrollment (comparing change in rate from pre-ADA to post-ADA)			Rate of Black/African American patient enrollment (evaluating change in rate in pre and post ADA periods)		
	IRR	95% CI	*P*	IRR	95% CI	*P*	IRR	95% CI	*P*	IRR	95% CI	*P*
Time (year)	1.01	1.00-1.02	0.27	1.01	1.00-1.02	0.27	1.13	1.05-1.22	< 0.01	1.13	1.05-1.22	< 0.01
ACA initiation	0.96	0.89-1.03	0.27	0.96	0.89-1.03	0.27	1.34	0.93-1.94	0.11	1.34	0.93-1.94	0.11
Change in rate of patients enrolled per year after ACA implementation	0.99	0.97-1.01	0.37	1.00	0.98-1.02	0.85	0.83	0.74-0.92	0.001	0.93	0.86-1.01	0.10

IRR = incidence rate ratio; CI = confidence interval

## Highlights

- Decreased enrollment of Black patients in high-risk breast cancer clinic post-ACA expansion
- Persistent racial disparities in enrollment in a high-risk breast cancer clinic
